# Emotionally expressed voices are retained in memory following a single exposure

**DOI:** 10.1371/journal.pone.0223948

**Published:** 2019-10-17

**Authors:** Yoonji Kim, John J. Sidtis, Diana Van Lancker Sidtis

**Affiliations:** 1 Department of Communicative Sciences and Disorders, New York University, New York, NY, United States of America; 2 The Nathan Kline Institute for Psychiatric Research at Rockland Psychiatric Center, Geriatrics Division, New York, NY, United States of America; 3 Department of Psychiatry, New York University Langone School of Medicine, New York, NY, United States of America; University of Hull, UNITED KINGDOM

## Abstract

Studies of voice recognition in biology suggest that long exposure may not satisfactorily represent the voice acquisition process. The current study proposes that humans can acquire a newly familiar voice from brief exposure to spontaneous speech, given a personally engaging context. Studies have shown that arousing and emotionally engaging experiences are more likely to be recorded and consolidated in memory. Yet it remains undemonstrated whether this advantage holds for voices. The present study examined the role of emotionally expressive context in the acquisition of voices following a single, 1-minute exposure by comparing recognition of voices experienced in engaging and neutral contexts at two retention intervals. Listeners were exposed to a series of emotionally nuanced and neutral videotaped narratives produced by performers, and tested on the recognition of excerpted voice samples, by indicating whether they had heard the voice before, immediately and after a one-week delay. Excerpts were voices from exposed videotaped narratives, but utilized verbal material taken from a second (nonexposed) narrative provided by the same performer. Overall, participants were consistently able to distinguish between voices that were exposed during the video session and voices that were not exposed. Voices experienced in emotional, engaging contexts were significantly better recognized than those in neutral ones both immediately and after a one-week delay. Our findings provide the first evidence that new voices can be acquired rapidly from one-time exposure and that nuanced context facilitates initially inducting new voices into a repertory of personally familiar voices in long-term memory. The results converge with neurological evidence to suggest that cerebral processes differ for familiar and unfamiliar voices.

## Introduction

How are new voices transformed into familiar voices? The traditional view of voice acquisition assumes that voice acquisition occurs incrementally with increased exposure to a particular voice. Based on this assumption, acquisition of voices has been studied in laboratory settings, where listeners were familiarized with the voices of selected speakers through repetitive training sessions [1, 2]. Although these “trained-to-familiar voices” [3] (p. 216) studies have value in examining underlying processes of voice perception and acquisition, their approach is limited in providing satisfactory explanations of familiar voice acquisition in naturalistic settings, where people may acquire and store a voice pattern through minimal but significant social encounters. The present study was designed to simulate naturalistic voice learning situations where voices are acquired—identified as having been heard before—through brief exposure when they are embedded in engaging, nuanced social and emotional contexts.

Based on one-trial learning mechanisms [4], the current paper tested the strong hypothesis that voices can be acquired as personally familiar, i.e., endorsed as having been heard previously, without frequent exposure, and further, the presence of nuanced, emotionally engaging context facilitates the acquisition of a new “familiar” voice. It was further hypothesized that this emotional underlay would lead to consolidating the voice into a repertory of known voices in long-term memory. The terms “emotional” and “emotional contexts” are used in this communication as surrogates for the more engaging, arousing condition, commanding attention, contrasted with the neutral condition, where less arousal and attention are inspired. By introducing emotional and neutral contexts into a voice recognition protocol, we manipulated the influence of nuanced, emotional context to evoke engagement, attention and arousal in listeners.

Specifically, listeners were exposed to videotaped narratives produced spontaneously with predominantly nuanced and emotional or neutral and dispassionate expression and, using audio excerpts, subsequently tested on recognition of recorded excerpts from companion narratives (same speaker, different narrative) both immediately and after one week. For the purpose of this paper, the term ‘familiar voices’ refers to voice patterns which have been acquired into a mental repertory of known voices, operationally defined here as endorsing a previous exposure. The design was intended to simulate (in the laboratory) conditions in real life, whereby one experiences a voice in a mutually engaging setting, such as a conversation, a setting that engages attentional mechanisms; this is intended to compare with hearing a voice, for example, which is backgrounded, spoken between two strangers, unattended to, or otherwise not personally engaging [3]. The natural setting typically includes visual material, particularly the face, with included facial expressions and head movements. Hand gestures were not included in the videotaped presentation. We use the term “emotionally expressed voice” as a surrogate for the personally engaging condition, and the term “neutrally expressed voice” for the indifferently experienced condition. In the experimental setting utilized here, successful recognition of a previously heard voice is proposed to constitute a beginning state of acquiring a personally familiar voice. In this study, the operational definition of “familiar voice recognition” utilizes the measure of subjects’ endorsing “having heard this voice before.” Our intention in this study was to reveal the very earliest glimmer of this process, proposing that “uptake” into a familiar voice repertory can occur quickly, with the assistance of engaging and affective nuance, presumed to be more effective in attracting attentional mechanisms. We have also used the terms “encoding” and “consolidation” of voice information, to clarify the often complex, vague connotations of the term “familiar.” Drawing on the one-trial learning mechanisms, the current study utilizes a single exposure, which is operationally defined as a one-minute exposure to a voice.

### Familiar voice recognition

Command over a large repertory of personally familiar voices can readily be verified throughout biology, in humans by introspection and in many other animals by empirical measures. Recent research has uncovered a great variety of astounding feats of auditory mastery in the form of individual voice recognition, across many species including frogs, who appeared on earth 250 million years ago [5]. In other examples, maternal seals return from a 6-month period of foraging for food to identify their pups by voice [6–10]. Penguin parents recognize the vocal signature of a single offspring in a wind-swept cacophony of continuous calling by hundreds of thousands of their peers [11,12]. Nonhuman primates recognize the voices of members of their extended family in a troop; 88 unique vocal patterns, carrying personal identity information, have been documented in the “known” voice repertories of baboons [13].

The ability to recognize significant others by voice has a meaningful and important presence in humans. Biological studies have revealed that newborns can recognize their mother’s voice even before birth and the newborn brain responds differentially to the maternal versus a “stranger” female voice [14]. The unique status of familiar voices has been supported by a variety of empirical studies indicating that recognizing familiar voices involves cognitive and neurophysiological processes that are distinct from those involved in perceiving and identifying unfamiliar voices [3, 15–19]. Despite the empirical and clinical importance of familiar voices, an upper limit for a typical human cache of familiarized voices has not yet been established [20], and specifically related to our study, there is little commentary to be found on how familiar voice patterns are naturally acquired throughout the human adult life span.

### Emotional memory

A large body of research suggests that emotionally significant events tend to be better remembered than events of less significance. This has been corroborated in numerous laboratory studies, using a variety of stimuli encompassing words [21, 22], film clips [23], and pictures [24]. These findings support the hypothesis that emotional information acquires salience and leave traces in our mind, and are thus more likely to be converted into long-term memory.

Memory enhancement for emotional events has been further discussed in the context of memory consolidation. Newly encoded memories are gradually stabilized over time, and finally integrated into pre-existing knowledge, a process known as memory consolidation [25, 26]. Memory consolidation involves two distinct stages: the cellular and molecular events that change synaptic connections over several minutes or hours following learning (known as “cellular consolidation”), and the reorganization of brain systems that occurs for an extended period of days and weeks following learning (known as “systems consolidation”) [27].

Converging evidence suggests that emotional aspects of memories are selectively consolidated over neutral aspects [28, 29]. This has been consistently shown in studies that assessed memories at various delays ranging from one week to several months following initial encoding (e.g., [21, 23, 30, 31]). These studies have demonstrated that memory for emotional experiences is enhanced following a period of consolidation, while relatively neutral experiences, encoded at the same time, do not receive such benefit. Building on evidence from prior studies [21], the current study used a time frame of a 1-week delay to allow for the process of memory consolidation.

Recent studies have shed light on an active role of sleep in consolidating memories [32, 33]. A study by Cairney et al. [33] showed that sleep strengthens both original and distorted memories following retrieval-induced distortion (RIB). Sleep has also been found to enhance implicit constraint learning and its generalization [32]. In addition, other studies detail retention of emotional memories after sleep [34–36]. For example, Payne et al. [35] focused on the effect of sleep on emotional memory trade-off, suggesting that sleep promotes memory for emotional scenes at the expense of neutral backgrounds. These findings provide compelling evidence that the brain differentially modulates information during sleep by selectively gating emotional and neutral components of memories.

Based on the literature and our intuition, the present study attempts to draw parallels between the process of personally familiar voice acquisition and emotional learning. Specifically, the process of a new voice taking on familiar status is comparable to that of information acquiring salience through arousal and emotional engagement. In emotional learning, information is selectively remembered and consolidated, when it is credited as personally relevant through an emotional experience. Likewise, a previously unfamiliar voice may be readily inducted into the known voice repertory and stored in long-term memory, when the voices are experienced in conditions that engage arousal and attention mechanisms. In contrast, a voice unattended to, and therefore not imbued with these contextual nuances may not be remembered, remaining “unfamiliar” [37]. We have used emotionally expressive versus neutrally expressed contexts to represent these two naturalistic states. The close relationship between emotionality and familiarity has been documented in person perception studies, in which emotionally salient stimuli are perceived to be more familiar than stimuli with less emotional significance [38, 39]. Both positive and negative emotions have shown to increase a sense of familiarity [40]. A study by Van Lancker and Ohnesorge [41], whereby famous persons and places were each rated on emotionality and familiarity scales, revealed a significant correlation between the two parameters.

### The current study

The current study investigated whether brief exposures to context-imbued voices influence the strength of the subsequent memory and its consolidation. In order to accomplish these goals, the current study was guided by the following research questions:

1. Does exposure to a voice for one minute suffice for acquiring a voice as recognizable—as having been heard before—when the voices occur in naturalistic settings?
2. Are voices with nuanced, emotional context acquired and stored preferentially in memory compared to voices experienced in neutral, dispassionate context?
3. Is memory of voices heard with engaging, emotional expression retained or stronger than that of neutrally expressed voices following a period of memory consolidation (i.e., one week after exposure)?

It was predicted that 1-minute exposure to a voice presented by a speaker in a videotaped narrative would allow for acquisition into memory of voices expressed in emotional and neutral spoken presentation, such that recognition performance on voices that had been heard (exposed/target voices) will be significantly higher than on voices that they had not heard (unexposed/foil voices). Given the potential role of engaging and emotionally expressive context on voice acquisition, it was further hypothesized that voices produced in an emotional context would result in better recognition, as compared with voices produced with relatively neutral expression. We also predicted that the emotional memory enhancement effect would persist or become greater after a delay. In particular, recognition for nuanced, engaging, emotionally expressed voices was expected to improve over time, whereas recognition for neutrally expressed voices was expected to decrease or remain unchanged over time.

## Methods

**Fig 1:**
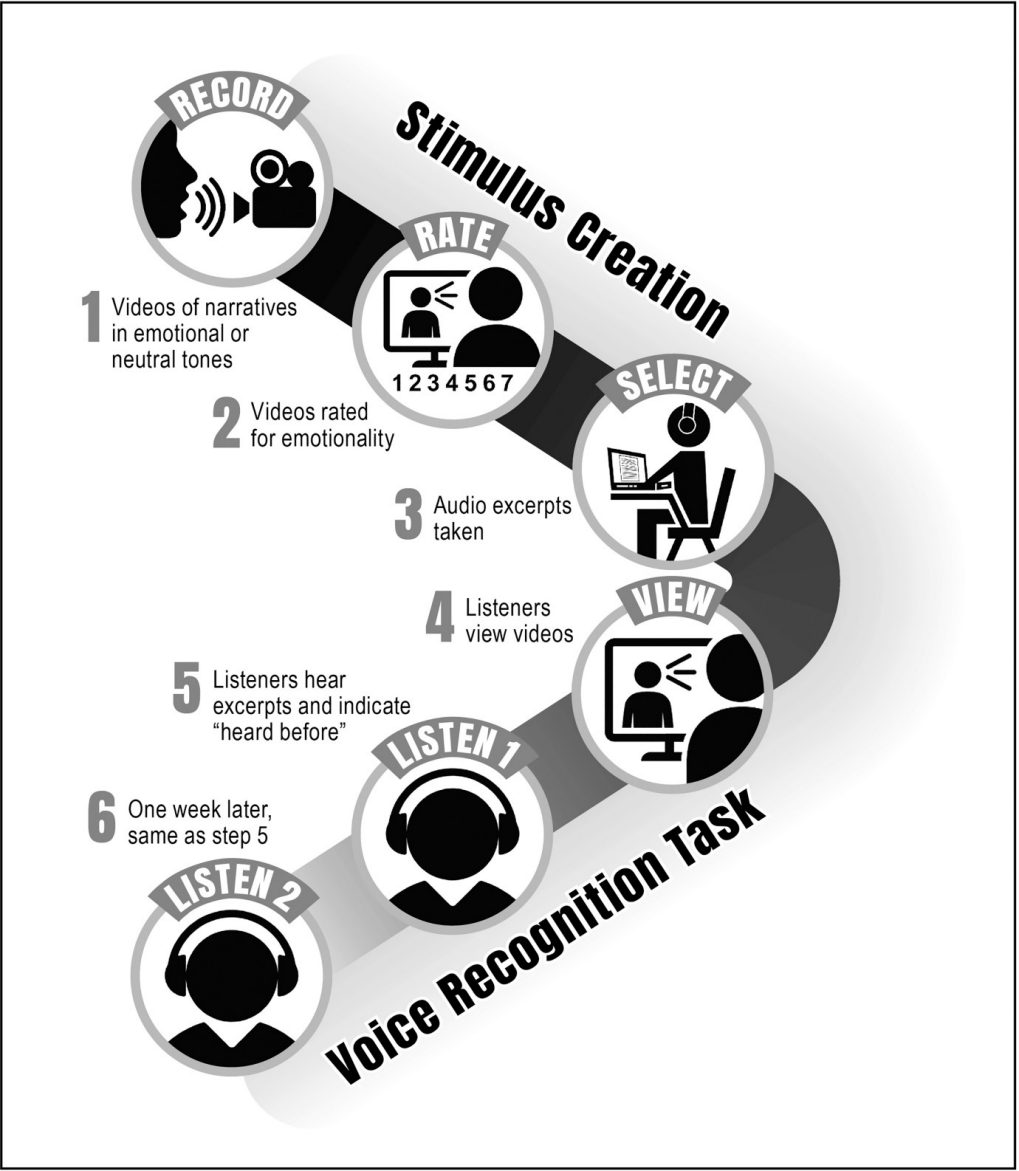
Flow chart of the experimental design representing the creation of the stimuli and the voice recognition tasks.

The voice recognition task was designed to investigate the effects of emotional context on encoding and consolidation of voices by testing participants immediately and one week after they were exposed to the target voices in videotaped presentations. The first session consisted of an exposure phase, during which listeners were exposed to target voices through videotaped narratives. Videos representing head shots were employed; these included facial expressions and head movements, as is encountered in a naturalistic situation. This was followed by a subsequent test phase, during which the participants were tested to recognize (as having heard before) voices of audio excerpts taken from companion videos for each speaker and control videos for additional new speakers. Test voices were taken from companion videos (videos produced by the same speaker but having different verbal content) for each narrator to probe actual voice recognition and not memory for specific lexical items in the excerpts. For the test phase, listeners’ task was to decide if the voice had been heard before (“yes,” if previously heard during exposure or “no,” if never before heard) and indicate how confident they felt about their responses on a 5-point scale ranging from “not at all confident” to “very confident.” In the second test session, given one week later, the same participants repeated the recognition test with no additional exposure to video narratives. A flow chart of the experimental design is shown in Fig 1 to represent the creation of stimuli and the voice recognition tasks.

The current approach does not endeavor to discover how listeners associate names with voices; instead, it investigates the ability to recognize having heard a voice at a previous time. In terms of emotional valence, the current study uses unspecified valence of emotion (positive or negative) to induce emotional engagement.

### Participants

Thirty-five native speakers of American English (26 females and 9 males; mean age = 26 years, range = 19–60 years; SD = 8.2 years) initially served as listeners. The sample size was determined based on prior experience with similar studies (38 participants in [1]; 30 participants in [42]), and availability of participants. All participants were born and raised in the United States, had learned English from birth, and reported no hearing difficulties. Participants were seen on two separate occasions, with a 1-week interval between sessions. The final sample consisted of 26 participants with 9 dropouts due to not returning for a second visit (n = 2), not meeting the eligibility criteria (n = 4), a technical error (n = 1), and failing to follow instructions (n = 2). Participants were allowed to adjust the volume to their comfortable listening levels in the beginning of the experiment. This volume was then maintained all the way through the end. The study was approved by the Institutional Review Board at New York University. All participants in the study provided written informed consent.

### Stimuli

The stimuli were audio and video recordings of emotionally and neutrally expressed narratives spontaneously produced by twenty-eight female performers (i.e., narrators) in a school for improvisational comedy. All narrators were native English speakers between the ages of 20 and 40 years with normal speech and hearing. Gender, age, and dialect are primary cues to voice perception. Therefore, to provide a homogeneous set, the narrators recruited for videotaping were restricted to younger female voices speaking General American. Our interest was to establish veridical voice recognition, not perception of vocal characteristics, as discernable in gender, age, or dialect features. Using both male and female voices, or a broad age range or variety of dialects, would confound the causal relationship between mode (emotional vs. neutral) and retention of voices. Further, it would be unclear whether the better retention of certain voices is caused by utilizing cues for a specific gender, age, or dialect. By selecting this set of relatively homogeneous voices, we were able to exclude the spurious effect of vocal characteristics on voice retention and ensure that higher recognition of voices produced in the engaging, emotional style is due to the manipulation of the mode (see review in Kreiman & Sidtis [3], Chapter 4).

Each narrator was asked to choose 2–4 topics to talk about before the video camera for 2–3 minutes each (see S1 Fig). Narrators could use fictional material and were allowed to select topics of their own. Suggested topics were marriage, birthday parties, travel, and holiday gatherings. Narrators were not coached on linguistic content or affective valence loading (positive or negative), but were alternately instructed to talk either in an “animated, expressive, nuanced, emotional” way or in a “neutral, calm, dispassionate, nonexpressive” way (see S1 Protocol). Therefore, approximately half of the narrators were randomly assigned to talk with heightened emotional expressiveness, and the other half to talk using neutral execution. These modes were perceptible in face, head movement, and voice; it was intended in these recordings for expressive context to be overall more or less engaging and arousing to the video viewer. This format was selected to simulate as far as possible a natural encounter with a person, whose voice was being experienced, with accompanying facial expressions and head movements. All other variables were held constant: all performers were recorded as head shots, all were instructed to talk about a topic of their choice; all spoke spontaneously and freely until asked to stop. Each performer produced 2–3 different video sessions. Following these procedures, eighty-five video clips were obtained and transcribed manually. Video files were edited using a video editing software, called iMovie. For the subsequent rating and recognition test, a display resolution of the videos was fixed at 960 × 540 pixels.

In order to select videotaped narratives that represented extremes of emotionally engaging and neutral expression, five female English-speaking adults were asked to rate the emotionality and level of engagement of eighty-five videos on two 7-point scales. The first rating scale represented emotional expressiveness as heard in voice, intonation, prosody and melody of speech in narrative on a continuum from “neutral” (1-very little or no emotional expression) to “expressive” (7-high emotional energy). The second 7-point scale rated how engaging the performer was in her narrative with 1 representing “not at all engaging” and 7 representing “very engaging” (see S2 Protocol).

The two ratings from each rater were averaged together for each video clip to choose videos from binary extremes. For the experiments, twenty-four videos (8 for Set A + 8 for Set B + 8 for control = 24), which received either the highest or the lowest mean ratings, were chosen to be used as stimuli. The number of videos from either extreme (emotional or neutral) was balanced in each set, resulting in a total of 12 emotional (4 for Set A + 4 for Set B + 4 for control) + 12 neutral (4 for Set A + 4 for Set B + 4 for control) = 24 videos. For the purpose of this study, half, taken from the emotional extreme, were classified as emotional and the other half, taken from the neutral extreme, were classified as neutral.

Using video recordings selected from the rating procedure, three sets of narratives were constructed: Set A, Set B, and Set C. Each set consisted of 8 videotaped narratives respectively. The prepared video narratives averaged 184 words in length with average durations of one minute (see S1 Text). Narratives in Set A and Set B were matched for the same speakers, but contained different narratives, representing exposed voices-unexposed excerpts. Therefore, during the exposure phase, participants viewed videotaped narratives from either Set A or Set B. The two exposed voice sets (A & B) were alternated in blocks across subjects. Narratives in Set C were produced by strangers and served as controls. Thus, narratives in Set C were not initially exposed to any of the participants before the recognition test and were used only as control audio excerpts for the testing.

Audio excerpts were taken from a companion narrative of each speaker (same voice, different verbal content) for the recognition test. This was done to ensure that voice recognition would not be assisted by verbal content. All excerpts were adjusted to average 70dB in intensity. Each excerpt was 10–15 words and averaged approximately 3–4 seconds long in the auditory form (see S2 Text). These audio excerpts were played from Praat with a sampling rate of 48kHz and 16-bit digitization. Half of the excerpts were taken from emotional narratives and half from neutral narratives. In an effort to minimize experimenter bias, excerpts made up of 10–15 words were selected in an evenly distributed fashion throughout each narrative. Specifically, five excerpts were taken from each narrative, resulting in a total of 40 (20 emotional + 20 neutral, either from Set A or Set B) + 40 (20 emotional + 20 neutral from Set C) = 80 excerpts for each participant. In other words, half of the excerpts represent voices that have been heard before (i.e., exposed voices-unexposed excerpts, or target voices, with a veridical answer of “yes” to the question “Have you heard this voice before”) and half represent voices that have not been heard before (i.e., foil voices, with a veridical answer of “no” to the same question). Excerpts were randomized according to four conditions: Emotional-Exposed, Emotional-Unexposed; Neutral-Exposed, Neutral-Unexposed. Exposure blocks A or B were alternated across subjects, in order to reduce any possible effects of particular excerpts or particular voices.

### Procedure

Each participant was tested individually in two sessions separated by one week. The first session lasted approximately 1 hour and the second session lasted approximately 0.5 hours. In the first session, each listener gave informed written consent and completed questionnaires about their language background (see S3 Protocol) prior to the experimental session. The participants were seated in a quiet room with a laptop computer in front of them. They were given all instructions and stimuli on 13-inch Macintosh computers and wore SONY MDR-7502 headphones during the task.

The first session involved two phases: exposure phase, and recognition testing phase. All listeners started with the exposure phase lasting approximately 9 min. They were told that they would watch videotaped stories narrated by female speakers and voice samples drawn from the speakers would be presented for the subsequent voice recognition test (see S4 Protocol).

During the exposure phase, each participant watched 8 videotaped narratives on the computer screen. The videotaped narratives were divided into two blocks of 8 narratives each, and thus each participant watched narratives either from Set A or Set B. Within each block, half of the narratives were spoken with emotional expression (4 emotional target voices) and half with neutral expression (4 neutral target voices). These were pseudo-randomized for presentation to the participants, with the order of E(Emotional)-N(Neutral)-N-E-N-E-E-N, which was the same for each participant. Each narrative was presented for 1 minute, with an inter-stimulus interval of 4 s. Blue screens were inserted between the videotaped narratives to signal a transition to the next narrator.

Immediately after the exposure phase, the test phase commenced, starting with 2 practice trials of listening to audio excerpts. Participants were given practice trials to acquaint themselves with the task procedure. No feedback was provided during practice trials and practice trials were not included in the analysis. Participants were then presented with 80 trials. On each trial, an audio excerpt was presented for 3–4 sec and the participants were asked to indicate their response by clicking with a mouse on one of two response options-“Yes” or “No” at the computer prompt, “Have you heard this voice before?” Half of the audio excerpts (n = 40) were target voices (i.e., exposed voices-unexposed excerpts), for which participants were expected to click a button marked “yes,” and the other half (n = 40) were foil voices (i.e., unexposed voices), for which the expected response was “no.” At the same time, half of the audio excerpts (n = 40) were emotional and half (n = 40) were neutral, such that each participant was exposed to four possible conditions of stimuli (i.e., emotional-exposed, emotional-unexposed, neutral-exposed, neutral-unexposed voices).

Following the “yes/no” classification, participants were asked to rate their confidence in their classification response by choosing a corresponding number on a scale from 1–5 (1 = “not at all” to 5 = “very”) on the computer screen. After providing a confidence rating, the participants were presented with the next audio excerpt in the series. This process continued until participants had heard, made a yes-no recognition judgment, and provided a confidence rating for each audio excerpt in the series. The order of excerpts was randomized, with each participant receiving the same sequence of excerpts. (The confidence ratings were not analyzed in the current study.)

At the end of the first session, participants were reminded that they were required to attend a second session after one week (mean = 7.15 days, range = 6–10 days). They were not informed that the second session would be a memory test. When participants returned for their second session, they were instructed to perform the recognition test as they did in the first session with no additional exposure phase. The second session was identical to the first except that, unlike in the first session, participants did not watch videotaped narratives (so that they were not additionally exposed to the target voices) and were not given practice trials prior to the test phase (see S5 Protocol).

## Results

According to signal detection theory [43], we calculated the measures of sensitivity (*d-prime*), and memory bias (*C*). D-prime was calculated by subtracting the z-score for the false alarm rate from the z-score for the hit rate, as follows: d' = z(hit rate)–z(false alarm rate). C was determined with the following equation: -0.5[z(hit rate) + z(false alarm rate)]. Statistical analyses were performed using SPSS software (IBM Corp., Armonk, NY, USA).

### Sensitivity *(d’)* and response bias *(C)*

The mean sensitivity measure *d’* was 0.6 across participants. A one-sampled t-test demonstrated that this *d’* was significantly greater than zero, *t*(25) = 10.4, *p* < .000. This result indicates that listeners were able to discriminate between exposed (target) and unexposed (foil) voices.

A repeated measures analysis of variance (ANOVA) conducted on the d-primes revealed a main effect of mode, with higher sensitivity to emotional voices than to neutral voices, *d’* = .77 vs. .43, *F*(1, 25) = 7.49, *p* = .011, η^*2*^ = .23 (Table 1). These findings indicate that recognition of emotional voices was overall more accurate than neutral voices. Participants were more likely to retain memory for emotional voices than memory for neutral voices. There was no significant main effect of time, *F*(1, 25) = .874, *p* = .359, nor interaction between mode and time, *F*(1, 25) = .013, *p* = .91.

**Table 1:** Mean and standard error for sensitivity measure *d’* as a function of mode and time.

		Mean	S.E.
Mode	Emotional	.77	.07
	Neutral	.43	.07
Time of test	Immediate	.56	.07
	Delayed	.64	.08

We also examined changes in the modulation of response bias by conducting repeated measures ANOVA on the bias measure *C* (Table 2). There were no main effects of mode, *F*(1, 25) = 3.59, *p* = .07, or time, *F*(1, 25) = 2.2, *p* = .15 or interaction of mode with time, *F*(1, 25) = 3.3, *p* = .083. These results suggest that there was no evidence that the listeners had a bias toward responding *yes* or *no* between the immediate and delayed conditions for either emotional or neutral voices.

**Table 2:** Mean and standard error for bias measure *C* as a function of mode and time.

		Mean	S.E.
Mode	Emotional	-.42	.06
	Neutral	-.31	.07
Time of test	Immediate	-.30	.05
	Delayed	-.43	.08

To further determine whether recognition memory for emotional voices changes over time, we examined the sensitivity and bias measures on the immediate and delayed tests separately. A series of paired samples t-tests were performed for each time condition, comparing performance between emotional and neutral voices. These tests were planned comparisons, made despite the lack of significant interactions. Table 3 presents a summary of these measures in the emotional/neutral and immediate/delayed conditions.

**Table 3:** Mean and standard errors of hit (HR) and false-alarm rates (FAR), *d'*, and *C* as a function of mode (emotional/neutral), and time (immediate/delayed) for 26 participants.

	Immediate				Delayed			
	Emotional		Neutral		Emotional		Neutral	
Measure	*M*	*SE*	*M*	*SE*	*M*	*SE*	*M*	*SE*
HR	.73	.03	.67	.03	.79	.03	.68	.03
FAR	.48	.02	.53	.03	.55	.03	.53	.04
*d*'	.72	.10	.40	.10	.81	.10	.46	.11
*C*	-.32	.07	-.29	.07	-.53	.09	-.34	.12

### Immediate test

**Fig 2:**
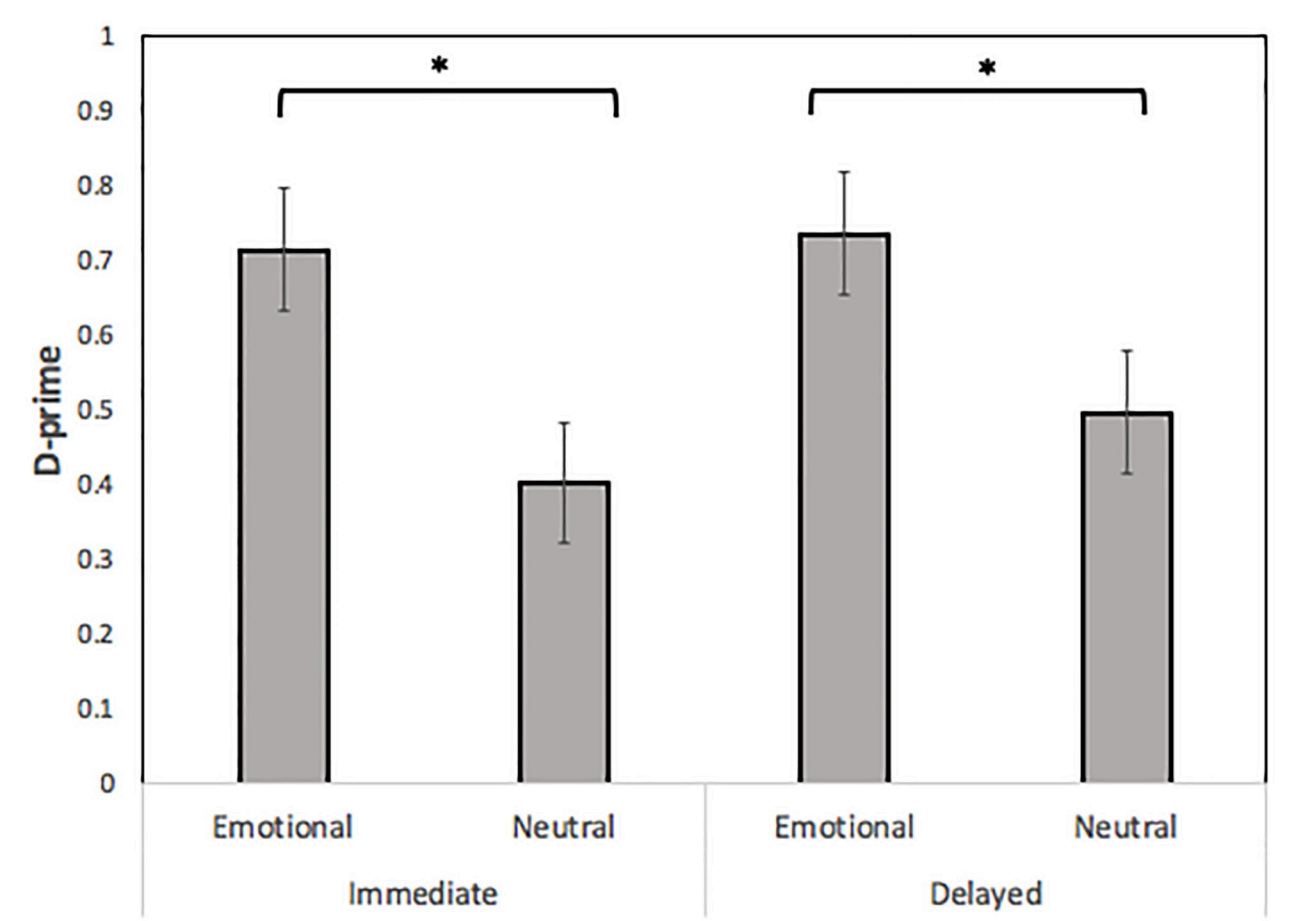
D-prime as a function of mode (emotional/neutral) and time (immediate/delayed). Error bars represent standard error of the mean. Significant differences are denoted by asterisks, *p < .05.

A paired samples t-test indicated that *d’* was significantly higher for emotional voices than for the neutral voices, *d’* = .72 vs. .4, *t*(25) = 2.12, *p* = .04, indicating that recognition of emotional voices was higher than for neutral voices (Fig 2). In an analysis of *C*, there were no significant differences in recognition bias between emotional and neutral voices, *t*(25) = -.41, *p* = .687.

### Delayed test

As during the immediate test, *d’* was again significantly higher for emotional voices than for the neutral voices, *d’* = .81 vs. .46, *t*(25) = 2.38, *p* = .025, which reflects the fact that recognition for emotional voices was still higher than it was for neutral voices after a 1-week delay (Fig 2). No significant differences in the bias measure *C* emerged, indicating that participants did not change their decision criteria in response to emotional or neutral voices, *t*(25) = -1.92, *p* = .067.

### Additional analyses

#### Serial position effect (primacy and recency)

Using accuracy measure, we further investigated whether primacy or recency effects occurred in the recognition test. Performance on excerpts taken from the first four videos was compared with performance on excerpts taken from the last four (of 8) videos for the immediate and delayed recognition test. Paired sample t-tests indicated no significant differences in accuracy on excerpts taken from the first half compared with the second half of the video exposure session for either the immediate or delayed recognition testing session, indicating that the video exposure session did not yield measurable primacy or recency effects in subsequent excerpt testing.

#### Order effects

In addition to randomizing test excerpts according to four conditions across the 80 recognition trials and organizing exposure to voices into two complementary blocks (A & B), which were alternated with listening groups, we performed another test of possible order effects. To further ensure that there was no inadvertent bias in the ordering of the conditions, the recognition accuracy for the first 10 excerpts was compared to the recognition accuracy for the last 10 excerpts for the immediate and the delayed tests. Performance on the first and last excerpts was not significantly different at either the immediate (*p* = .646) or delayed recognition (*p* = .456) tests.

#### Effect of linguistic content on recognition memory

Analysis was performed to ascertain a possible contribution of linguistic content (here, referring to words) to enhanced memory for emotional materials. Specifically, percentages of emotional words were compared in all the emotional vs neutral narratives presented during the exposure phase, and emotional vs neutral excerpts presented to listeners. (Please note that the excerpts were taken from companion narratives, spoken by the same performer, but not included in the video-narrative exposure experience.) In order to obtain the percentage of emotional words, a text analysis software called Linguistic Inquiry and Word Count (LIWC) was used [44]. The LIWC, developed as a tool for quantitative research in studies of cognition and emotion, provides a percentage of words of a given category by matching words in a target text to a dictionary. For the purpose of the current study, among a variety of categories offered by LIWC, a category that taps into emotion, called 'affect' was utilized. Each narrative or excerpt was loaded into LIWC to determine the percentage of words devoted to 'affect' category.

A paired sample t-test on proportions of emotional words revealed no significant difference between emotional narratives (*M* = 6.1, *SD* = 2.4) and neutral narratives (*M* = 4, *SD* = 2.2) [*t*(7) = 1.65, *p* = .14, two-tailed]. Similarly, a paired t-test examining excerpts revealed no significant differences in proportions of emotional words between emotional (all two tailed) (*M* = 5.9, *SD* = 7) and neutral exposed excerpts (*M* = 4.7, *SD* = 6) [*t*(39) = 0.82, *p* = .42]; between emotional exposed (*M* = 5.9, *SD* = 7) and emotional control (*M* = 4.6, *SD* = 5) [*t*(39) = .99, *p* = .33]; or between neutral exposed (*M* = 4.7, *SD* = 6), and neutral control (*M* = 5, *SD* = 6.6), [*t*(39) = -.15, *p* = .88].

## Discussion

The present study offers evidence of an effective impact of emotionally expressive context on familiar voice acquisition, although revealing only the earliest stage of this important process. The study design utilized emotionally expressed, nuanced narratives to stand for (simulated in the experimental setting) naturalistic, personally relevant interaction with another talker, in contrast with neutrally expressed vocal material intended to represent voices heard in less personally engaging conditions [45]. To answer the first question, whether listeners are able to acquire a voice as familiar following a one-minute, one-time encounter, the current study suggests that the answer is positive. The one-sample t-test conducted on the d' confirms the listeners’ ability to reliably recognize voices that were exposed to very briefly. Listeners made accurate recognition judgments based on voices they heard (and saw) for one minute. This result suggests that brief exposure to a voice communicates enough information for the listener to uniquely and efficiently remember having heard the voice. The second and third questions related to whether emotional vs. neutrally expressive voices are differentially encoded, and whether such disparity persists or increases across a delay. Our findings demonstrate that listeners revealed greater sensitivity in the responses made to emotional voices compared to neutral voices in both immediate and delayed tests. In other words, the enhanced memory for emotional voices was statistically significant when tested immediately and a week after the exposure. These findings indicate that emotional expressiveness, theoretically inducing arousal and engagement in the listener, was shown to be a catalyst for transferring new voices into a “recognized identified as known” voice repertory. Our finding that the memory enhancement effect for emotionally experienced voices was salient even when tested immediately suggests that the recognition advantage of emotional over neutral voices does not necessarily require the passage of time. These observations are compatible with the findings of the previous studies by Talmi and her colleagues [46, 47], suggesting that the emotional memory enhancement effect can appear in the immediate tests and be maintained across the delay. It is our belief that this result reflects the very beginning of the process of uploading a voice into a natural repertory of personally familiar voices.

Using LIWC, a program to quantify emotional words in discourse, no differences were found between emotional and neutral exposed narratives; nor were any significant differences in proportions of emotional words seen in comparing neutral and emotional excerpts in exposed or control conditions. This serves to exclude an effect of differential proportion of emotional words in the stimulus material as possibly influencing the results.

In our experiment, participants were successful in recognizing voices when they heard new excerpts of the exposed voices (i.e., exposed voices-unexposed excerpts; stimuli did not constitute the same verbal, linguistic material to which listeners had been previously exposed). These findings suggest that participants were able to transfer the underlying voice patterns to novel utterances, and that this transfer was mediated by implicit knowledge acquired during the exposure phase. Overall performance did not diminish over time as might be expected from previous work. For example, a study by Legge, Grosmann and Pieper [48] showed that learning 20 unfamiliar voices was challenging to listeners, but once those voices were inducted into familiar voice repertory, listeners were able to remember them even after 10 days. These findings reflect the implicit nature of the retention of a potentially familiarized voice.

Furthermore, the improved recognition of voices in an emotional context provides further evidence that the facilitative effect of emotionally expressive context on voice acquisition was not limited to the excerpts that participants actually heard, but it was generalized to new excerpts of the previously exposed voices. Successful retention of the voice pattern occurred after one minute of exposure to the face and the voice, a format that was intended to simulate naturalistic experiences of acquiring new voices in everyday life. It is clear that in natural life, some voices are acquired without visual information, for example, from radio listening. Our design does not predict results from this kind of scenario. A further experiment might determine the efficiency of auditory-only exposure.

This study provides the first piece of experimental evidence that recognition of a previously heard voice can occur from a single, one-minute exposure to a narrative setting including voice and face with the support of emotional, nuanced context, and the specified quality of exposure to the voice, not just the quantity, contributes to transferring a new voice to a personal repertory of familiar voices. To speculatively extrapolate the findings to a model of veridical familiar voice acquisition, it might be said that a single personally relevant encounter with a speaking person leads to potential retention of the voice pattern, such that subsequent exposure to the voice has a feeling of familiarity, which may then be further consolidated into a stronger memory trace. A previous study revealed an effect of interactive, personal conversation on retention of a voice pattern, presumably engaging arousal and attention in the conversation participant, in comparison to passive listening [49]. In another study, naturally produced voices, intended to imitate conversational dialogue, were found to be successfully recognized in some conditions [50].

Similarly, recent research by Reuterskiöld and Van Lancker Sidtis [51] demonstrated that children learned idiomatic expressions rapidly following a single exposure in naturalistic settings, in which they were actively engaged in meaningful conversations. Acquisition of idioms, like acquiring a unique voice pattern, is also characterized by selective attention and arousal. These findings lend support to a view that engaging, meaningful, and socially interactive experiences are more likely to lead to significant learning, enabling rapid uptake of information with single exposure. Extreme examples of this process have been amply described under the rubric of “flashbulb memory” [52–54]. The present experiments introduced emotional context into an audiovisual task, which, although involving passive watching and listening, presumably also engaged arousal and attention in the listener, resulting in superior storage of a memory trace of the voice in the emotional context, which was retained after a delay, as compared to neutral context.

Evidence from clinical and empirical studies points to a psychological distinction between familiar voice recognition and unfamiliar voice perception or discrimination [15, 18, 55–57]. Clinical studies have identified persons with dissociations between these two kinds of competence following focal brain damage [58, 59]. Familiarity with a voice aids speech perception [60, 61] and ERPs are enhanced by a familiar voice stimulus [62]. In contrast, unfamiliar voices occur unnoticed in the surround, even when changed during an interaction (change deafness) [37, 63, 64]. The study reported here supports this view of special status for the familiar voice in cognition.

Our findings highlighting the preferential consolidation of emotionally expressive voices are supported by converging evidence from neuropsychological and neuroimaging studies, showing the influence of emotional arousal on memory [65, 66]. According to contemporary accounts of human emotions [67], brain circuits located deep in the subcortical system are referred to as “motivational centers”; emotionally salient or motivationally relevant stimuli (appetitive or aversive) automatically elicit heightened attention [68–70]. Enhanced perceptual encoding for emotional voices, as observed in our study, might be attributed to the participants’ increased attention directed toward those voices. Further, neurological studies point to a right hemisphere involvement in recognizing familiar voices [71, 72] as well as modulating the familiarity of objects, faces, persons, and locations and emotional experiencing [19, 73]. Limbic and right hemisphere systems provide a welcoming substrate for this kind of process. Introducing a new voice into membership in a repertory of personally familiar voices likely engages these several brain systems. This cerebral configuration highlights the important differences between the processing of familiar and unfamiliar voices as revealed in neurological studies [58].

In sum, the current study reveals the potency of a single, one-minute voice exposure, especially when accompanied by nuanced conditions, in the process of storage to memory. Further, this study adds to the existing literature by highlighting a role of emotion as a facilitator of long-term memory retention and extending this evidence to voice perception. Results obtained in this study suggest that emotional engagement plays a critical role in facilitating the process of a new voice pattern ascending into a familiar repertory in long-term memory. The basis for the effect of brief exposure on voice recognition appears to rest in human’s remarkable capabilities for recognizing voices and storing large amounts of voice information in memory. These prodigious voice recognition abilities might have arisen in our evolutionary past nearly 250 million years ago, much earlier than the emergence of language [74], implying that the ability to recognize voices relevant to the species’ particular biological function has high survival value, and affords evolutionary advantages.

## Supporting information

S1 Fig Stylized screenshot example of video. (PDF)

S1 Text Examples of transcribed narratives. (PDF)

S2 Text Examples of audio excerpts. (PDF)

S1 Protocol Instructions for recording. (PDF)

S2 Protocol Instructions for emotionality ratings. (PDF)

S3 Protocol Language background questionnaire. (PDF)

S4 Protocol Instructions for immediate recognition task. (PDF)

S5 Protocol Instructions for delayed recognition task. (PDF)
