# Supplementary material for: Emotionally expressed voices are retained in memory following a single exposure
Source: PLoS One. 2019 Oct 17;14(10):e0223948. doi: 10.1371/journal.pone.0223948 (PMC6797471; doi:10.1371/journal.pone.0223948)
Supplement: S1 Fig — (PDF) [file pone.0223948.s001.pdf]

**S1 Figure. Stylized screenshot example of video**

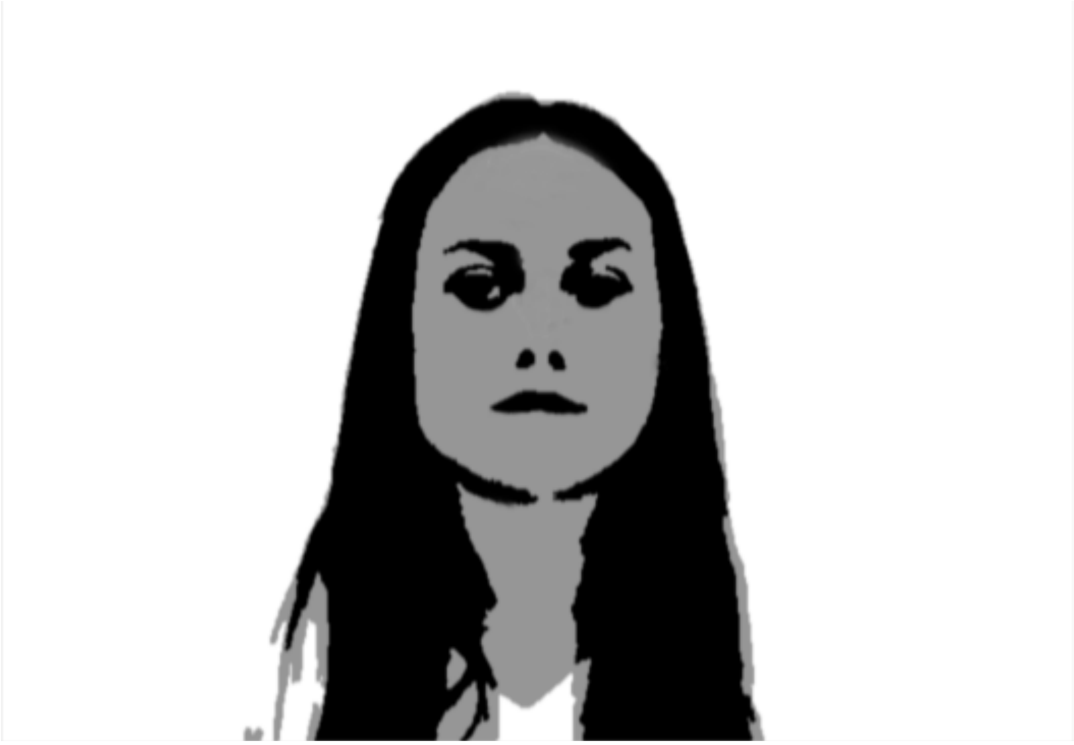

The image has been altered to obscure the identity of the participant.
