# Supplementary material for: Emotionally expressed voices are retained in memory following a single exposure
Source: PLoS One. 2019 Oct 17;14(10):e0223948. doi: 10.1371/journal.pone.0223948 (PMC6797471; doi:10.1371/journal.pone.0223948)
Supplement: S1 Text — (PDF) [file pone.0223948.s002.pdf]

S1 Text. Examples of transcribed narratives

Emotional narrative

...which is like the most exciting thing cause it's like he's gonna be there. Us uh – ultimately that's like all that like matters is like is that he's there and anybody else who doesn't like anything can leave. But like I'm so excited because I don't know. We get to like do all of this stuff. And and I've sort of made up for last time like getting on the Pinterest and and looking up all this stuff. And like you know we're about to write vows. That's kind of terrifying. Cause I I feel like his vows are gonna be better than my vows. His vows are gonna be so much better than mine. And I'm gonna be like I love you, you know, cause I like freeze up. I freeze up with – I can be angry really easily. But like showing like love I get like I get nervous cause I'm not that kind of person. Like on you know whatever... but yea I'm just excited. And then the engagement was amaze – see he's just like – it makes me like - I'm so happy, but he's so amazing. He's so amazing. And I just like can never, I can never meet up to his level because like even our engagement.

Neutral narrative

...a lot of equipment that I help manage. Uh I actually just got a job here as an equipment manager, so I take care of all the cameras. I make sure they're in good shape. As well as our lighting equipment, and our audio equipment. Um it's been great learning about how different equipment works. And I really feel like I've learned a lot and grown a lot in my job. Um, and sometimes on the side I work with some of my friends here and we make little films, uh, on our off time. Uh it's been really great to be part of a community that has such creative people here. It's been a lot of fun... Right now I'm working on a series with a friend, uh, about break ups. And we're exploring – and it's a comedic series - so we're exploring different breaks ups in different neighborhoods of New York. And she plays all the characters. Um, it's been really really great to film that...
