# Supplementary material for: Emotionally expressed voices are retained in memory following a single exposure
Source: PLoS One. 2019 Oct 17;14(10):e0223948. doi: 10.1371/journal.pone.0223948 (PMC6797471; doi:10.1371/journal.pone.0223948)
Supplement: S2 Text — (PDF) [file pone.0223948.s003.pdf]

S2 Text. Examples of audio excerpts

Excerpts taken from emotional narratives

1. my dad was like “Oh I have this map. We’ll just follow this map
2. the middle of nowhere, we finally see another car. and my moms like
3. my dad. She still gets angry when she talks about this.
4. me. and we go and I write down. And we’re just sitting in the car
5. all I wanted was to be home by the time the game started and we
6. So last week I got cast in a new ... show at the
7. And luckily I got the younger sister princess who is- she’s bored
8. And when I went to the audition I went in and I took
9. my song which was normally a love song if you would sing it normally in
10. it’s sort of a love song about letting someone go. But instead of

Excerpts taken from neutral narratives

1. Um...I’m hoping that when we get there someone will give us a
2. far away from that world for so long, you kinda remem, like you don’t
3. you have to take. So hopefully someone will show us
4. uh the pictures look really beautiful. I’m particularly excited about
5. let us use those little glow lights so that we can see our
6. a lot of my friends’ little sisters and little brothers graduating from high
7. And it’s brought back memories from when I graduated. I
8. a really great day. I mean, my best friends were there.
9. in college, so we were able to graduate together. And
10. family was there. And it’s one of those days where you just feel
