# Supplementary material for: Emotionally expressed voices are retained in memory following a single exposure
Source: PLoS One. 2019 Oct 17;14(10):e0223948. doi: 10.1371/journal.pone.0223948 (PMC6797471; doi:10.1371/journal.pone.0223948)
Supplement: S1 Protocol — (PDF) [file pone.0223948.s004.pdf]

S1 Protocol. Instructions for recording

For the recording procedure, narrators were randomly assigned to talk in either emotional tones or neutral tones. The following instructions were given to the narrators assigned to the emotional condition.

Thank you for participating our voice recognition study.

EXPRESSIVE VOICES
Please talk for 2-3 minutes about two topics of your choice, consecutively, choosing from the list provided below. You can also suggest topics of your own which you would like to talk about. Please talk into the microphone and look at the video camera.
Your story-telling should use highly expressive intonation and melody of speech. Please tell your story in a manner that is animated, energetic, and inspired.

- *Your background and your career choice (what inspired you to become an actor)*
- *Birthday party*
- *Boyfriend/Girlfriend/Spouse*
- *Wedding*
- *Car ride*
- *Family*
- *Graduation*

The following instructions were given to the narrators assigned to the neutral condition.

Thank you for participating our voice recognition study.

NEUTRAL VOICES
Please talk for 2-3 minutes about two topics of your choice, consecutively, choosing from the list provided below. You can also suggest topics of your own which you would like to talk about. Please talk into the microphone and look at the video camera.
Please tell your story in neutral, easy tones, using a calm, deliberate manner of delivery. Your story-telling should avoid expressive intonation of any kind.

- *Your background and your career choice (what inspired you to become an actor)*
- *Birthday party*
- *Boyfriend/Girlfriend/Spouse • Wedding*
- *Car ride*
- *Family*
- *Graduation*
