# Supplementary material for: Emotionally expressed voices are retained in memory following a single exposure
Source: PLoS One. 2019 Oct 17;14(10):e0223948. doi: 10.1371/journal.pone.0223948 (PMC6797471; doi:10.1371/journal.pone.0223948)
Supplement: S2 Protocol — (PDF) [file pone.0223948.s005.pdf]

S2 Protocol. Instructions for emotionality ratings

The following instructions were provided to the raters.

Please watch the videos in the order they appear. We would like you to perform two ratings on each video.

The first is the emotional expressiveness as heard in voice, intonation, prosody and melody of speech in story telling. Your ratings will be placed on a continuum from "neutral" (1-very little or no emotional expression) to "expressive" (7-high emotional energy).

The second is a rating of how engaging the performer is in her story telling, again on a scale where 1="not at all engaging" and 7="very engaging."
Please take care to match the header of the video with the label on the rating sheet.
