# Supplementary material for: Emotionally expressed voices are retained in memory following a single exposure
Source: PLoS One. 2019 Oct 17;14(10):e0223948. doi: 10.1371/journal.pone.0223948 (PMC6797471; doi:10.1371/journal.pone.0223948)
Supplement: S3 Protocol — (PDF) [file pone.0223948.s006.pdf]

S3 Protocol. Language background questionnaire

The following represents an example of the language background questionnaire.

Intake Sheet

* Start:

Finish:

Subject Number: ______________________________

Age: ______________________________

Date: ______________________________

Years of Education: ______________________________

Language spoken since birth: ______________________________

Second Language: ______________________________

Gender: ______________________________

Major or Profession: ______________________________

Where tested: ______________________________
