# Supplementary material for: Emotionally expressed voices are retained in memory following a single exposure
Source: PLoS One. 2019 Oct 17;14(10):e0223948. doi: 10.1371/journal.pone.0223948 (PMC6797471; doi:10.1371/journal.pone.0223948)

S4 Protocol. Instructions for immediate recognition task

In the exposure phase, the following instructions were provided to the listener-participants on the computer screen before the videotaped narratives were presented.

Thank you for participating in this study. First, you will view eight brief videotaped stories narrated by female speakers. Next, voice samples drawn from the speakers will be presented as auditory stimuli on the computer.

Immediately after the videos were screened, the following statement appeared on their screen:

Now your task is to respond with "Yes" or "No" at the computer prompt, to indicate whether or not you recognize the voice of each voice sample and indicate the level of confidence on a scale of 1 (not at all) to 5 (very). We will begin with 2 practice items.

The following figure shows an example of the displays that appeared on the participants' screens during practice and experimental trials.

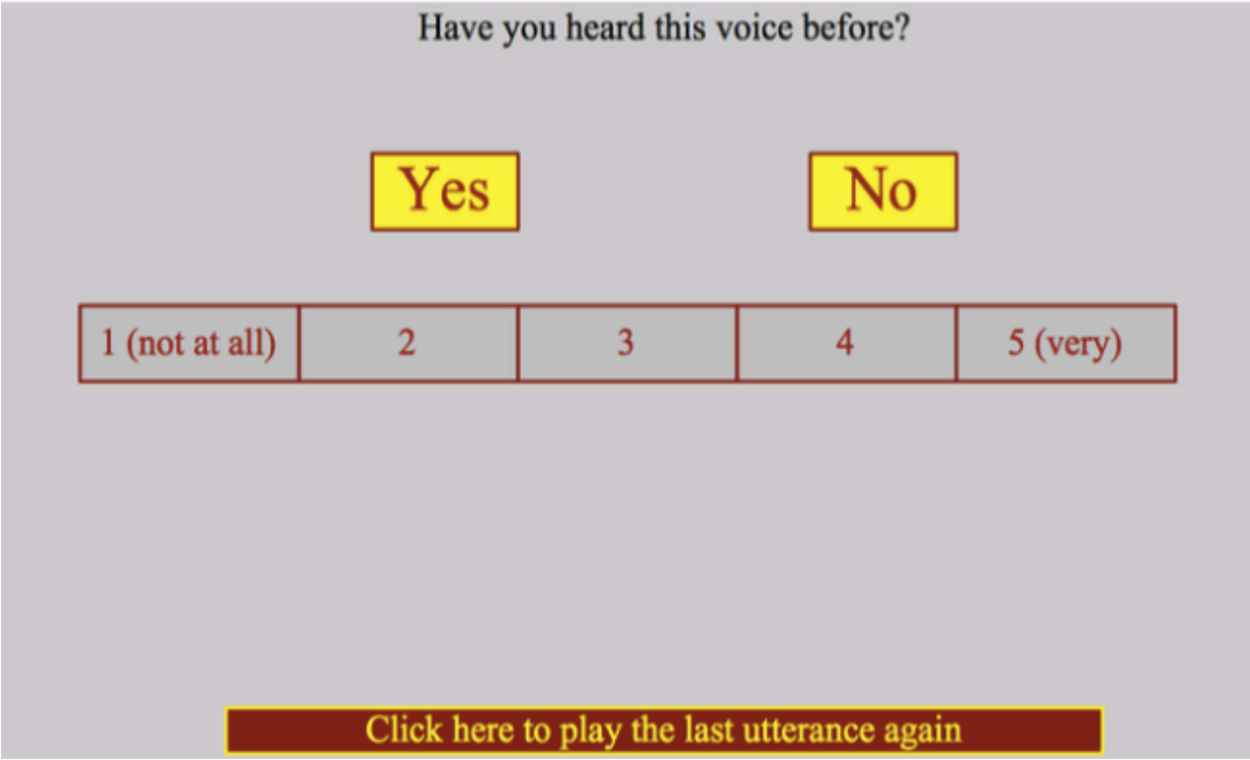

Supplement: S4 Protocol — (PDF) [file pone.0223948.s007.pdf]
