# Supplementary material for: Emotionally expressed voices are retained in memory following a single exposure
Source: PLoS One. 2019 Oct 17;14(10):e0223948. doi: 10.1371/journal.pone.0223948 (PMC6797471; doi:10.1371/journal.pone.0223948)
Supplement: S5 Protocol — (PDF) [file pone.0223948.s008.pdf]

S5 Protocol. Instructions for delayed recognition task

For the delayed recognition task, the following statement was presented on a computer screen prior to the test.

Thank you for your continued participation in our study. We would like to test your memory for the voices that you had heard in the first session. You will hear voice samples drawn from the speakers. As you performed last time, we would like you to respond with "Yes" or "No" at the computer prompt, to indicate whether or not you have heard the voice before. Then, we would like you to rate how confident you feel about the responses you have provided on a scale of 1 (not at all) to 5 (very).
